# Insulin Adherence and Associated Factors in Patients with Type 2 Diabetes Mellitus Treated in Klang Primary Health Care Centres

**DOI:** 10.21315/mjms2021.28.6.8

**Published:** 2021-12-22

**Authors:** Nasruddin AZRI, Bachok NORSA’ADAH, Norul Badriah HASSAN, Nyi Nyi NAING

**Affiliations:** 1 Unit of Biostatistics and Research Methodology, School of Medical Sciences, Universiti Sains Malaysia, Kubang Kerian, Kelantan, Malaysia; 2 National Pharmaceutical Regulatory Division, Ministry of Health Malaysia, Petaling Jaya, Selangor, Malaysia; 3 Department of Pharmacology, School of Medical Sciences, Universiti Sains Malaysia, Kubang Kerian, Kelantan, Malaysia; 4 Faculty of Medicine, Universiti Sultan Zainal Abidin, Medical Campus, Kuala Terengganu, Terengganu, Malaysia

**Keywords:** type 2 diabetes mellitus, insulin, injections, medication adherence, primary health care

## Abstract

**Background:**

Insulin therapy is necessary for patients with type 2 diabetes mellitus (T2DM) to reach the targeted glycaemic level and prevent complications. This study aimed to determine the proportion of adherence to insulin therapy and the associated factors in patients with T2DM.

**Methods:**

A cross-sectional study was conducted among 249 patients with T2DM who had been on insulin therapy for at least 2 months in primary care centres of the Ministry of Health in Klang, Malaysia. A validated insulin adherence questionnaire for diabetes mellitus (DM) was used to assess insulin adherence. Data on the sociodemographic characteristics, disease-related factors, treatment-related factors and clinical parameters were extracted from medical records and interviews with patients.

**Results:**

The adherence to insulin therapy was 8.43%. The factors associated with insulin adherence were self-monitoring of blood glucose (SMBG) (adjusted odds ratio [AOR]: 5.39; 95% confidence interval (CI): 1.20, 24.13; *P* = 0.028), exercise (AOR: 3.38; 95% CI: 1.37, 10.03; *P* = 0.029) and the number of daily insulin injections (AOR: 1.63; 95% CI: 1.09, 2.44; *P* = 0.017).

**Conclusion:**

The adherence to insulin therapy in primary health care centres in Malaysia was very poor. Patients who practiced SMBG, exercised and frequent daily insulin injections were significantly more adherent to insulin therapy.

## Introduction

Type 2 diabetes mellitus (T2DM) is a common chronic disease worldwide and its trends are increasing year after year (1). Diabetes mellitus (DM) caused 1.5 million deaths worldwide in 2012 and contributed to an additional 2.2 million deaths due to the increased risks of cardiovascular disease and other complications (2). It is estimated that by 2035, 592 million of the world’s population would have DM, and large proportions of them live in low and middle-income countries (3).

The National Health Morbidity Survey, which is conducted routinely by the Ministry of Health Malaysia, reported an increasing trend in the prevalence of T2DM from 6.3% in 1986, 8.3% in 1996, 11.6% in 2006, 15.2% in 2011, 17.5% in 2015 and is expected to increase to 20.6% in 2020 (4). In 2015, 17.6% of people aged 40 years old–44 years old had DM, compared to 10.3% in 2006 (4). Those aged 45 years old–49 years old experienced an increase of 15%–20.6% in the same year (4).

Uncontrolled T2DM is characterised by hyperglycaemia which leads to various complications and considerably affects the quality of life of individuals and the risk of premature death. Ischaemic heart disease, cerebrovascular events, nephropathy, retinopathy, peripheral neuropathy and leg amputation are complications of diabetes (2). Twenty to forty percent of patients with DM develop kidney disease and subsequently, end-stage renal disease (2). The prevalence of diabetic nephropathy was 54.3%, according to a study conducted in a Malaysian hospital (5). A scientific review of 18 studies reported improved glycaemic control in T2DM delayed or prevented long-term complications (6).

Diabetes was poorly controlled among patients attending public hospitals in Malaysia. The percentage of diabetic patients under optimum control was only 13% in tertiary centres and 24% in primary care centres (7). Data from online Adult Diabetes Control and Management from 303 centres of 70,889 T2DM reported only 30.9% attained haemoglobin A_1c_ (HbA_1c_) less than 7.0% (8).

Most patients with T2DM would ultimately require insulin therapy as an advance option to maintain good blood sugar control. Even though T2DM patients are recommended for insulin therapy, they often resist physician recommendations, in part because of misconceptions about injectable medication (9). A large cross-sectional study of Malaysian with T2DM reported only 10% of patients were treated with insulin (8). A review of 34 qualitative studies on the perceptions of insulin therapy in the management of T2DM reported three categories of multifactorial barriers (10). The first is the patient-centred factors which include insulin-related beliefs, social influences, psychological factors, hypoglycaemia and therapy barriers; the second is factors related to the clinician which include insulin skills of general practitioners, integration of healthcare, healthcare professional-perceived barriers, hypoglycaemia and explanations of compliance; and the third is the healthcare professional-patient relationship factor from the perspectives of patients and clinicians. Patient-related barriers have been reported to account for approximately 30% of factors contributing to resistant insulin therapy. These barriers to patients depend on a variety of factors, such as health literacy, costs, number of medications, trust in their physician, communication and time with their physician (11).

Once insulin is prescribed, patients must comply with the regiment satisfactorily. The patient must follow many aspects of insulin therapy. One of these is self-monitoring of blood glucose (SMBG), which is crucial for dose adjustment and has been associated with improved control of T2DM. Adherence to pharmacological treatment is still unsatisfactory and is a serious concern, especially in the population of developing countries like Malaysia. A study of diabetic patients in a tertiary centre on the east coast of Malaysia showed that the proportion of adherence to insulin therapy among Malay ethnicity was 19%, which was considered unsatisfactory (12). The reasons for non-adherence are multifactorial. Adherence to insulin therapy was influenced by advanced age (13), sex (13), presence of comorbidities (13), duration of diabetes (14), number of concomitant medications (15), adverse effects (1, 16) and duration of insulin therapy (14, 17).

Studies in different settings with different types of population samples regarding race, culture, lifestyle, education and income could yield different results. It has never been found that a study using a comprehensive questionnaire on all aspects of insulin compliance in diabetic patients. The common methods of measuring insulin adherence in diabetic patients have been reported to include the medication possession ratio, proportion of days covered, persistence, average daily consumption and the Morisky Medication Adherence Scale (MMAS) (18). The systematic review concluded that the methods mentioned were not a quality measure for insulin adherence and the need for a new measurement tool. Therefore, the present study aimed to determine adherence to insulin therapy using a new questionnaire and its associated factors in T2DM patients treated in government primary care clinics in Klang, Selangor.

## Methods

### Study Design

This was a cross-sectional study. We included respondents with T2DM aged 18 years old and above, had been on insulin therapy for at least 2 months, and were able to understand and read the Malay language questionnaire. Respondents with T1DM and gestational diabetes, unwilling to participate, could not communicate well with the interviewer, on the first visit to the health facility and who had incomplete data records of more than 30% were excluded from this study.

The minimum required sample size was calculated using a single proportion formula with 19% insulin adherence (12), 5% precision, 0.05 alpha and a power of 80%. The minimum sample size required for this study was 263. All patients presented at the pharmacy’s counter were screened by reviewing their sociodemographic, medical history and medications to ensure that all inclusion criteria were met. Eligible patients were selected, identified and invited to participate in the study while waiting for their number to be called. If they agreed to participate, they were invited to a private room and the researcher then explained the informed consent. Convenient sampling method was applied. The respondents were given self-administered questionnaires, with the researcher being available for further explanation and clarification on the questions if needed. Respondents then, returned the completed questionnaire to the researcher.

### Study Location

This study was conducted in five health clinics located in the Klang district of Selangor state in Malaysia. Klang is the third largest district but the second most populous district in Selangor. The main ethnicity distribution in Klang district was 41% Malays, 27% Chinese and 21% Indian. Klang district was chosen because it is multi-racial, thus this study can generate a more general result on adherence to insulin therapy which can be inferred to the multi-racial population of Malaysia. The majority of Malaysian would utilise health care provided by the government. It has been reported that 60.1% of the Malaysian population preferred government health services for out-patient care (5). The utilisation of government out-patient services was higher for a rural location, ingenuous and Malays ethnicity, lower education and housewives or the unemployed. The reasons are related to the availability and cost of the services. Therefore, the adherence to insulin in this study could be inferred from the low socioeconomic status of Malaysian population.

### Research Tools

The data collection form was divided into three parts: i) sociodemographic details (age, gender, race, marital status, educational level, employment status and household income); ii) clinical characteristics (medical history and management of DM) and iii) validated Malay version of Insulin Adherence Questionnaire for Diabetes Mellitus (IAQDM) (12).

The IAQDM measures insulin compliance in the past 2 months. The questionnaires have 34 items in four domains: i) insulin compliance (8 items); ii) monitoring of insulin dose and sugar level (7 items); iii) self-adjustment of insulin therapy (9 items) and iv) problems of insulin injection (10 items). The IAQDM had been validated with good internal consistency with Cronbach alpha 0.88 (12). Subjects were asked to circle the option chosen in the 0 to 100 response scale with six categories. The categories are: i) 0 for ‘never’; ii) 10 and 20 for ‘rarely’; iii) 30 and 40 for ‘sometimes’; iv) 50 and 60 for ‘often’; v) 70 and 80 for ‘very often’ and vi) 90 and 100 for ‘all the time’. The total score of all items and its percentage were calculated. The percentage of the total score of ≥ 80% was considered adherence to insulin therapy (12).

T2DM diagnosis in this study refers to respondents with fasting blood sugar (FBS) of ≥ 7 mmol/L or random blood sugar (RBS) of ≥ 11.1 mmol/L or HbA_1c_ ≥ 6.3% (7). In this study, the cut-off point ≥ 7 mmol/L for FBS was considered high and < 7 mmol/L was considered normal. For RBS, the cut-off point ≥ 11.1 mmol/L was considered high while < 11.1 mmol/L was considered normal. Meanwhile, for HbA_1c_, the cut-off point of ≥ 6.3% was considered high and < 6.3% was considered normal. Only the last reading of glycaemic indexes was extracted from the medical records. Exercise refers to a minimum of 20 min of physical activity three times a week to develop and maintain physical fitness (19).

## Statistical Analysis

Data entry and analysis was conducted using Stata standard edition version 14. All continuous variables were expressed as mean and standard deviation (SD) or as median and interquartile range (IQR) depending on the distribution, while categorical variables were expressed in frequency and percentage. Binary logistic regression was used to identify the factors associated with insulin adherence. The dependent variable was dichotomous, namely adherence and non-adherence to insulin therapy. The independent variables were sociodemographic characteristics, disease-related characteristics and treatment-related characteristics. Univariable analysis was performed to screen for significant independent variables. The independent variables with a *P*-value of less than 0.25 in the simple logistic regression and those that are clinically important were considered to be included in the multivariable logistic regression. Forward and stepwise forward methods were used for variable selection. The principle of best fit, biologically plausible, clinically important and statistically significant were applied in the multivariable binary logistic regression by seeking the most parsimonious model. The crude and adjusted odds ratio (OR), regression coefficient, 95% confidence interval (CI), Wald statistic and *P*-value were presented.

## Results

### Sociodemographic and Clinical Chracteristics of Respondents

A total of 249 respondents were included in the final analysis. Male consisted of 53.6% of the total respondents. The mean of the normally distributed age of the respondents was 58.2 years old with SD 8.6. The majority of the respondents were Malay (45.0%), retired (39%), with secondary education (65.9%) and with monthly income above RM2,501 (61.5%). Ninety percent of respondents were non-smokers and respondents who exercised were 56.6%. The comparisons of sociodemographic characteristics of the respondents are presented in Table 1. There was no significant association between sociodemography variables and adherence to insulin therapy.

The mean of DM duration was 12.7 (SD 7.8) years. The record review showed that the mean number of clinic visits in the past year was 4.8 (SD 2.5) times. More than half of respondents reported that they had no family history of DM and had never been admitted into a hospital for DM in the past year. The majority of the respondents had no complications related to DM (74.7%) or comorbidity (91.2%). The mean and SD of the number of comorbidities was 2.4 (0.7) diseases. The disease-related characteristics of the adherence and non-adherence respondents are shown in Table 2. There was no significant association between disease-related factors and adherence to insulin therapy.

The mean number of concomitant medicines was 6.5 (SD 1.8), while the median duration of the use of insulin therapy was 3.0 (IQR 3.5) years. Five types of insulin therapy were prescribed, with 36.6% on basal insulin and 34.5% on pre-mixed insulin. The mean number of daily insulin injections was 2.1 (SD 1.1) and the mean total of daily insulin dose was 42.4 IU (SD 28.8). Most respondents self-injected their insulin (94.0%). Meanwhile, 35.3% had preference for oral therapy, 14.5% had fear of injection, 17.3% did use traditional and complementary medicine (TCM), 11.7% had experienced adverse effect of insulin therapy (hypoglycaemia and weight gain), 63.5% practiced SMBG and only 6.4% had attended the Medication Therapy Adherence Clinic (MTAC). The diabetic treatment of the adherence and non-adherence respondents are shown in Table 3. There were significant associations between the number of daily insulin injections (*P* = 0.014), the use of TCM (*P* = 0.049) and SMBG (*P* = 0.017) and adherence to insulin therapy. T2DM respondents who had good adherence to insulin therapy had significantly more daily injections, used more TCM and SMBG than those who had less adherence.

The majority (95.6%) of respondents had high HbA_1c_, 64.3% had a high FBS level and 31.1% had a high RBS level. The last blood glycaemic parameters of T2DM respondents are shown in Table 4. There was no significant association between blood glycaemic measurements and adherence to insulin therapy.

### Adherence Status and the Associated Factors

The proportion of adherence to insulin therapy was only 8.4% (95% CI: 6.0, 13.0). The final model for the associated factors of adherence to insulin therapy was presented in Table 5. A patient who practiced SMBG had 5.39 times the odds to adhere to insulin therapy compared to a patient who did not practice SMBG after adjusting for exercise and the number of daily insulin injections (adjusted OR [AOR]: 5.39; 95% CI: 1.20, 24.13; *P* = 0.028). A patient who exercised had 3.38 times the odds to adhere to insulin therapy compared to a patient who did not exercise after adjusting for SMBG and the number of daily insulin injections (AOR: 3.38; 95% CI: 1.37, 10.03; *P* = 0.029). There was a 63% increase in the odds of adherence to insulin per unit increase the number of daily insulin injections after adjusting for SMBG and exercise (AOR: 1.63; 95% CI: 1.09, 2.44; *P* = 0.017).

## Discussion

Although the majority of our respondents were Malay ethnicity, there was also adequate involvement of other major ethnic groups of Malaysian, mainly Chinese and Indian. The respondents had good social support as most of them were married and lived with family or spouse. Most of the respondents were retired, which is generally found in the older group. The majority of them had a monthly household income of more than RM2,501 per month and had completed at least secondary education.

Our study revealed that adherence to insulin therapy in the past 2 months was very low (8.4%). This finding was compared to 19% in a study that used the same questionnaires but of Malay ethnic in a tertiary referral centre in the Kelantan state (12). Our study was conducted in a multi-ethnic population who utilised healthcare services in primary care centres in a large district of one of the most developed states in Malaysia. It was well known that government primary health care is generally used by low socioeconomic and low education groups of the population (5). In comparison, the adherence to oral medications in T2DM was 47% in Malaysia (13). Oral medications are generally easier to comply with and have fewer barriers to administer than injectables. A study in Iran revealed that 28.8% of patients had adhered to insulin therapy (20), while a study involving 3,637 subjects in France reported a rate of adherence of 39% (21). A study in a tertiary centre in Brazil reported adherence of 17.6% to 27.8% to insulin according to the Morisky-Green questionnaire and an adherence of 30.6% to 41.7% based on the IAQDM (22).

A telephone survey in Turkey reported that 20.1% of patients withdrew and 59.6% adhered to insulin in the past 6 months (14). In addition, a study in the United States of America showed that adherence to hypoglycaemic agents, including insulin therapy, was 28% (23). The study was using MMAS’s scores, that 51% of patients were classified with high adherence (score of 0), 42% with medium adherence (score of 1–2) and 7% with low adherence (score of 3–4). The most common reasons for non-adherence were forgetfulness (39%) and carelessness (25%) (23).

There are great variations in the rate of adherence to the insulin; that were related to the methods, including definitions of insulin adherence, the time frame of adherence, measurement tools, type of healthcare and the population itself. A systematic review of 78 publications reported that none of the methods identified are perfect as a measure of the quality of insulin adherence in patients with diabetes (18). Each of the different methods for measuring insulin adherence has advantages and disadvantages, which must be considered when assessing their applicability as a quality measure (18). Self-report questionnaires are subject to reporting bias and patients tend to over-report their adherence (18). It was unlikely that our patients were over-reported the adherence, given the low percentage of results. Even though we were using self-reported questionnaires to measure adherence, we were using newly validated questionnaires that were developed specifically for our local population, and all aspects of insulin administration were measured by using 34-items psychometric measurements. Yavuz et al. (14) who reported 59.6% adherence, measured insulin adherence based on definition. Discontinuation of treatment after starting it was considered as non-persistence and skipping at least one injection of insulin in a week was considered non-adherence.

Our study found a non-significant association between adherence and glycaemic control, which was in contrast to other studies. A significant difference in HbA_1c_ was observed in patients from minimal adherence group compared to the moderate group and maximal group of MMAS questionnaire (24). Patients who had lower adherence to insulin therapy had higher HbA_1c_ levels than those with higher adherence. It was found that each one-point increase in MMAS total score was associated with an increase of 1.8 mmol/mol in HbA_1c_ measured 6 months later after adjusting for baseline HbA_1c_ (23).

Many factors have been found inconsistently as factors associated with medication adherence in T2DM patients. Our study revealed that the factors significantly associated with adherence to insulin therapy were all modifiable risk factors which are SMBG, exercise and the number of daily insulin injections. Our study found that a patient practicing SMBG had 5.39 times more likely to adhere to insulin therapy compared to a patient who was not practicing SMBG. Numerous studies have supported this finding, but Gomes and Negrato (24) found that SMBG was not related to adherence to insulin therapy. Diabetes is a disease that requires self-management that includes lifestyle modifications, self-monitoring of blood glucose as well as adjustment of insulin dosage. The SMBG should be used as a guide for adjusting therapy, thus allowing patients to be in control of their health (9). Patients who have had SMBG have adopted a positive self-management approach to their management of DM, as monitoring of their glycaemic levels enables them to notice the effect of insulin therapy on their glycaemic control and ultimately, lead to the better adherence to insulin therapy. Despite the benefit of the SMBG, only 3.4% of the 70,889 T2DM patients practiced it at home (8). A randomised controlled trial among uncontrolled T2DM studied the effect of home gluco-telemonitors that was designed to facilitate self-management to improve medication adherence, and encourage a healthier lifestyle and use of resources to reduce risk factors (25). It showed the intervention group was significantly better glycaemic control compared to the group that received routine healthcare service. Continuous and frequent glucose monitoring was associated with controlled glycaemic levels (26).

T2DM patients of our study, who exercised were 3.38 times more likely to adhere to insulin therapy than patients who did not exercise. Patients who exercised regularly are more health conscious and therefore, have better adherence to insulin therapy. Unfortunately, there is limited literature on the association between exercise and adherence to medication, or studies of physical activity and exercise in the local population with DM (27).

Our study also revealed that a 63% increase in the probability of adherence to insulin per one insulin injection a day. This finding was confirmed by Peyrot et al. (28) and Purran (12) who concluded that the number of daily injections significantly affects adherence to insulin therapy. However, Gomes and Negrato (24) found that the number of daily insulin injections was not significantly related to adherence to insulin therapy. A higher number of insulin injections would allow patients to be constantly vigilant to take their insulin injection and would therefore lead to greater adherence to treatment. A patient who needs three or four injections daily would likely be more aware of their efforts to take care of their overall health. The high number of injections also reflects uncontrolled diabetes; thus, they receive more advice that emphasises greater compliance.

Associated non-modifiable factors such as age, ethnicity, gender, employment status, educational level, income per month and smoking status were non-significantly related to adherence to insulin therapy in our study. These results were consistent with a study by Gomes and Negrato (24) which stated that gender and ethnicity were not related to adherence to insulin therapy. Regardless of this, male and Malay ethnic in our study were found to be more adherent to medication than female and other ethnics, respectively.

There was also no significant association between the presence of comorbidities related to DM and adherence to insulin therapy. Unlike previous studies that reported the presence of DM-related comorbidities significantly impacts the adherence to medication (13, 17). Our study also found that the duration of DM had no association with adherence to insulin therapy while other studies concluded that the longer duration of DM leads to poorer adherence to insulin therapy (14, 29). A longer duration of DM was associated with non-adherence to the recommendations of dietary changes and a higher probability of skipping an insulin dose (14).

The main strength of our study is the use of the validated Malay version IAQDM which specifically created to measure the adherence level to insulin therapy in the Malaysian population. Our study used a threshold of more than 80% as adherent and below 80% as non-adherent to insulin therapy and assessed the adherence for the past two months. In addition, we used an advanced statistical analysis of binary logistic regression which was specially adjusted for confounders and the detection of influential outliers was used in the analyses.

There are several limitations to this study. This study was a cross-sectional study design that measured only an association. The findings could be different or the results would be more informative if a longitudinal study were employed. In addition, the study was only conducted in one district in Selangor state. Our findings were based on a self-administered questionnaire. A self-reported method using a questionnaire could have been influenced by recall bias and the tendency of patients to provide satisfactory responses to researchers or social desirability. These biases can lead to underestimation or overestimation of adherence. However, the self-reported questionnaire has been widely used to measure the level of adherence as they are low in terms of cost and time and more importantly, could provide precise and reasonable estimates of the level of adherence. We suggest using several tools together to be more precise in measuring insulin adherence. A combination of self-administered questionnaires with pharmacological monitoring is reasonable. Despite all of these limitations, our study provides valuable and unique data for future studies.

We encountered several problems while collecting data. There are 15 health clinics in Klang district, that did not have an equal number of T2DM patients on insulin therapy. Some clinics had very few patients who met our selection criteria. We performed a convenient sampling due to time constraints. It is probably better to have an equal number of respondents from each of the health centres to be a better representative of the population of Klang.

More research is needed to understand better the factors that have contributed to the level of adherence to insulin therapy locally in Malaysia. Further research could explore and study other aspects that influence insulin therapy such as cost, attitude, emotional states, diet and lifestyle of patients. In addition, exploration of the psychological aspect of patients regarding problems related to injections could be useful. We suggest an in-depth study to discover the barriers that cause a low level of adherence. A qualitative study would explore in depth the barriers and obstacles of respondents.

The findings of our study showed that adherence to insulin therapy could be improved for many patients. As the number of patients attending government primary care centre continues to increase year by year due to improvement in the government health care system and increasing medical cost, the Ministry of Health Malaysia must take a proactive approach to address medication adherence issues among insulin therapy users. Focusing on the effort to improve patients’ understanding of the DM-related complications and the usefulness of insulin therapy in their DM-management would be a reasonable approach.

Primary care physicians play a challenging role in the management of T2DM patients by ensuring patients’ positive reception to insulin therapy. They must be able to address and overcome patient resistance and their own barriers to optimise insulin therapy. Communication between patient-physician is very important. The physician should listen to fears and concerns about insulin therapy and provide ongoing education and counselling to facilitate adherence to their treatments. Due to the low adherence, we suggest closer monitoring by the attending physicians. This monitoring can be done by paramedics as well as by physicians with an emphasis on individual self-management of diabetic treatment that include lifestyle modification, self-monitoring of glucose level and insulin dosage adjustment by the patients.

## Conclusion

Adherence to insulin therapy in the Klang district was poor as the proportion was 8.4%. Medication adherence is a fundamental component of self-management for T2DM patients undergoing treatment. The significant associated factors of adherence to insulin therapy were SMBG, exercise and the number of daily insulin injections. The associated factors identified in this study would assist policymakers and provide additional information to health care professionals to improve the adherence level to insulin therapy in government primary care clinics. This study was an important step in helping physicians and researchers to better understand the nature of adherence and to develop approaches to improve it. Furthermore, the findings of this study can be inferred to the population with similar characteristics.

## Figures and Tables

**Table 1:** Sociodemographic characteristics of T2DM respondents treated with insulin therapy (*n* = 249)

Variables	Frequency (%)		*P*-value*

	Adherence *n* = 21	Non-adherence *n* = 228	
Age (years old)†	59.4 (8.0)	58.1 (8.6)	0.524
Gender			
Female	8 (38.1)	108 (47.4)	0.417
Male	13 (61.9)	120 (52.6)	
Race			
Chinese	2 (9.5)	48 (21.0)	
Indian	8 (38.1)	79 (34.7)	0.274
Malay	11 (52.4)	101 (44.3)	0.223
Employment status			
Not working	8 (38.1)	77 (33.8)	
Retired	9 (42.9)	88 (38.6)	0.975
Employed	4 (19.0)	63 (27.6)	0.438
Education level			
No education	4 (19.1)	12 (5.3)	
Primary	2 (9.5)	19 (8.3)	0.221
Secondary	12 (57.1)	152 (66.7)	0.052
Tertiary	3 (14.3)	45 (19.7)	0.027
Monthly income (RM)			
< 1,000	6 (28.6)	62 (27.2)	
1,001–2,500	4 (19.1)	24 (10.5)	0.430
> 2,500	11 (52.3)	142 (62.3)	0.674
Smoking status			
Non-smoker	20 (95.3)	204 (89.5)	
Smoker	1 (4.7)	24 (10.5)	0.414
Exercise			
No	5 (23.8)	103 (45.2)	
Yes	16 (76.2)	125 (54.8)	0.067

Notes:

* simple logistic regression;

† mean (SD)

**Table 2:** Disease-related characteristics of T2DM respondents treated with insulin therapy (*n* = 249)

Variables	Frequency (%)		*P*-value*

	Adherence *n* = 21	Non-adherence *n* = 228	
Duration of DM†	12.4 (9.6)	12.8 (7.7)	0.849
Family history of DM			
No	8 (38.1)	54 (23.7)	
Yes	13 (61.9)	174 (76.3)	0.150
Hospital admission due to T2DM in past year			
No	20 (95.2)	201 (88.6)	
Yes	1 (4.8)	26 (11.4)	0.363
Presence of DM-related complications			
No	16 (76.2)	170 (74.6)	
Yes	5 (23.8)	58 (25.4)	0.870
Number of clinic visits in past year†	4.1 (1.9)	4.8 (2.6)	0.260
Presence of comorbidity			
No	1 (4.8)	21 (9.2)	
Yes	20 (95.2)	207 (90.8)	0.500
Number of comorbidity†	2.5 (0.7)	2.4 (0.7)	0.443

Notes:

* simple logistic regression;

† mean (SD)

**Table 3:** Treatment-related characteristics of T2DM respondents treated with insulin therapy (*n* = 249)

Variables	Frequency (%)		*P*-value*

	Adherence *n* = 21	Non-adherence *n* = 228	
Number of concomitant medications†	6.3 (1.3)	6.5 (1.9)	0.695
Duration of use of insulin therapy†	4.1 (3.8)	3.1 (2.4)	0.110
Type of insulin			
Prandial	1 (4.8)	13 (5.7)	
Basal	2 (9.5)	89 (39.0)	0.329
Pre-mixed	10 (47.6)	44 (19.3)	0.323
Combination	8 (38.1)	82 (36.0)	0.829
Number of daily insulin injections†	2.7 (1.2)	2.0 (1.1)	0.014
Total daily dosage of insulin (IU)†	52.6 (28.6)	41.4 (28.6)	0.093
Self-injection of insulin			
No	0 (0.0)	15 (6.6)	
Yes	21 (100.0)	213 (93.4)	0.623
Preference of oral therapy			
No	17 (80.9)	144 (63.2)	
Yes	4 (19.1)	38 (36.8)	0.113
Fear of injection			
No	20 (95.2)	193 (84.6)	
Yes	1 (4.8)	35 (15.4)	0.216
Use of TCM			
No	14 (66.7)	192 (84.2)	
Yes	7 (33.3)	36 (15.8)	0.049
Experience insulin’s side effect			
No	19 (90.5)	201 (88.2)	
Yes	2 (9.5)	27 (11.8)	0.752
SMBG			
No	2 (9.5)	89 (39.0)	
Yes	19 (90.5)	139 (61.0)	0.017
Attended MTAC			
No	20 (95.2)	213 (93.4)	
Yes	1 (4.8)	15 (6.6)	0.746

Notes:

* simple logistic regression;

† mean (SD)

**Table 4:** The last blood glycaemic levels of T2DM respondents treated with insulin therapy (*n* = 249)

Variables	Frequency (%)		*P*-value*

	Adherence *n* = 21	Non-adherence *n* = 228	
HbA_1c_			
Normal	2 (9.5)	9 (3.9)	
High	19 (90.5)	219 (96.1)	0.235
RBS†			
Normal	17 (80.9)	151 (67.7)	
High	4 (19.1)	72 (32.3)	0.210
FBS‡			
Normal	3 (18.7)	52 (37.7)	
High	13 (81.3)	86 (62.3)	0.135

Notes:

* simple logistic regression;

† 2% missing values (*n* = 244);

‡ 38.15% missing values (*n* = 154)

**Table 5:** The factors associated with adherence to insulin therapy among respondents with T2DM (*n* = 249)

Variables	Simple logistic regression			Multiple logistic regression		

	ß (SE)	Crude OR (95% CI)	*P*-value	ß (SE)	AOR (95% CI)	*P*-value
SMBG						
No	-	1	-	-	1	-
Yes	1.81 (0.76)	6.08 (1.38, 26.75)	0.017	1.68 (0.77)	5.39 (1.20, 24.13)	0.028
Exercise						
No	-	1	-	-	1	-
Yes	0.97 (0.53)	2.64 (0.93, 7.44)	0.067	1.22 (0.56)	3.38 (1.14, 10.03)	0.029
Number of daily insulin injections	0.47 (0.19)	1.60 (1.10, 2.32)	0.014	0.49 (0.20)	1.63 (1.09, 2.44)	0.017

Notes: b(SE) = Regression coefficient (standard error)

The linearity of number of daily insulin injections was performed and reported to be linear, multicollinearity and interaction were unlikely, the overall fit of the model was checked and reported Hosmer-Lemeshow test (*P* = 0.989), Pearson Chi-squared test (*P* = 0.589), Correctly classified table 91.57%, Area under the ROC curve 76.15% (*P* < 0.001)
